# Functional characterization of the *Mycobacterium abscessus* genome coupled with condition specific transcriptomics reveals conserved molecular strategies for host adaptation and persistence

**DOI:** 10.1186/s12864-016-2868-y

**Published:** 2016-08-05

**Authors:** Aleksandra A. Miranda-CasoLuengo, Patrick M. Staunton, Adam M. Dinan, Amanda J. Lohan, Brendan J. Loftus

**Affiliations:** 1 School of Medicine, Conway Institute of Biomolecular and Biomedical Research, University College Dublin, RmG043 Conway Institute, University College Dublin, Belfield Dublin 4, Ireland; 2 APC Microbiome Institute, Biosciences Institute, University College Cork, Cork, Ireland

**Keywords:** Integrated map, *Mycobacterium abscessus*, WhiB7, Antibiotic, Cystic fibrosis

## Abstract

**Background:**

*Mycobacterium abscessus subsp. abscessus* (MAB) is a highly drug resistant mycobacterium and the most common respiratory pathogen among the rapidly growing non-tuberculous mycobacteria. MAB is also one of the most deadly of the emerging cystic fibrosis (CF) pathogens requiring prolonged treatment with multiple antibiotics. In addition to its “mycobacterial” virulence genes, the genome of MAB harbours a large accessory genome, presumably acquired via lateral gene transfer including homologs shared with the CF pathogens *Pseudomonas aeruginosa* and *Burkholderia cepacia*. While multiple genome sequences are available there is little functional genomics data available for this important pathogen.

**Results:**

We report here the first multi-omics approach to characterize the primary transcriptome, coding potential and potential regulatory regions of the MAB genome utilizing differential RNA sequencing (dRNA-seq), RNA-seq, Ribosome profiling and LC-MS proteomics. In addition we attempt to address the genomes contribution to the molecular systems that underlie MAB’s adaptation and persistence in the human host through an examination of MABs transcriptional response to a number of clinically relevant conditions. These include hypoxia, exposure to sub-inhibitory concentrations of antibiotics and growth in an artificial sputum designed to mimic the conditions within the cystic fibrosis lung.

**Conclusions:**

Our integrated map provides the first comprehensive view of the primary transcriptome of MAB and evidence for the translation of over one hundred new short open reading frames (sORFs). Our map will act as a resource for ongoing functional genomics characterization of MAB and our transcriptome data from clinically relevant stresses informs our understanding of MAB’s adaptation to life in the CF lung. MAB’s adaptation to growth in artificial CF sputum highlights shared metabolic strategies with other CF pathogens including *P. aeruginosa* and mirrors the transcriptional responses that lead to persistence in mycobacteria. These strategies include an increased reliance on amino acid metabolism, and fatty acid catabolism and highlights the relevance of the glyoxylate shunt to growth in the CF lung. Our data suggests that, similar to what is seen in chronically persisting *P. aeruginosa*, progression towards a biofilm mode of growth would play a more prominent role in a longer-term MAB infection. Finally, MAB’s transcriptional response to antibiotics highlights the role of antibiotic modifications enzymes, active transport and the evolutionarily conserved WhiB7 driven antibiotic resistance regulon.

**Electronic supplementary material:**

The online version of this article (doi:10.1186/s12864-016-2868-y) contains supplementary material, which is available to authorized users.

## Background

The incidence of disease caused by *Mycobacterium abscessus* (MAB) is increasing and it is emerging as an important cystic fibrosis (CF) pathogen with a high innate antibiotic resistance rendering it refractory to most antimicrobial therapies [1]. Comparison of the genomes of multiple MAB strains have highlighted the presence of a large number of accessory genes likely acquired through horizontal gene transfer [2]. Little is known about the regulatory architecture of the genome however and functional genomic resources are scarce. We begin to address this deficit through the generation of a multi-omics map of MAB and the generation of a number of transcriptional datasets in response to important clinically relevant stresses.

## Results & discussion

### An integrated approach combining RNA-seq/Ribo-seq and Proteomics identifies the coding potential of MAB

**Fig. 1:**
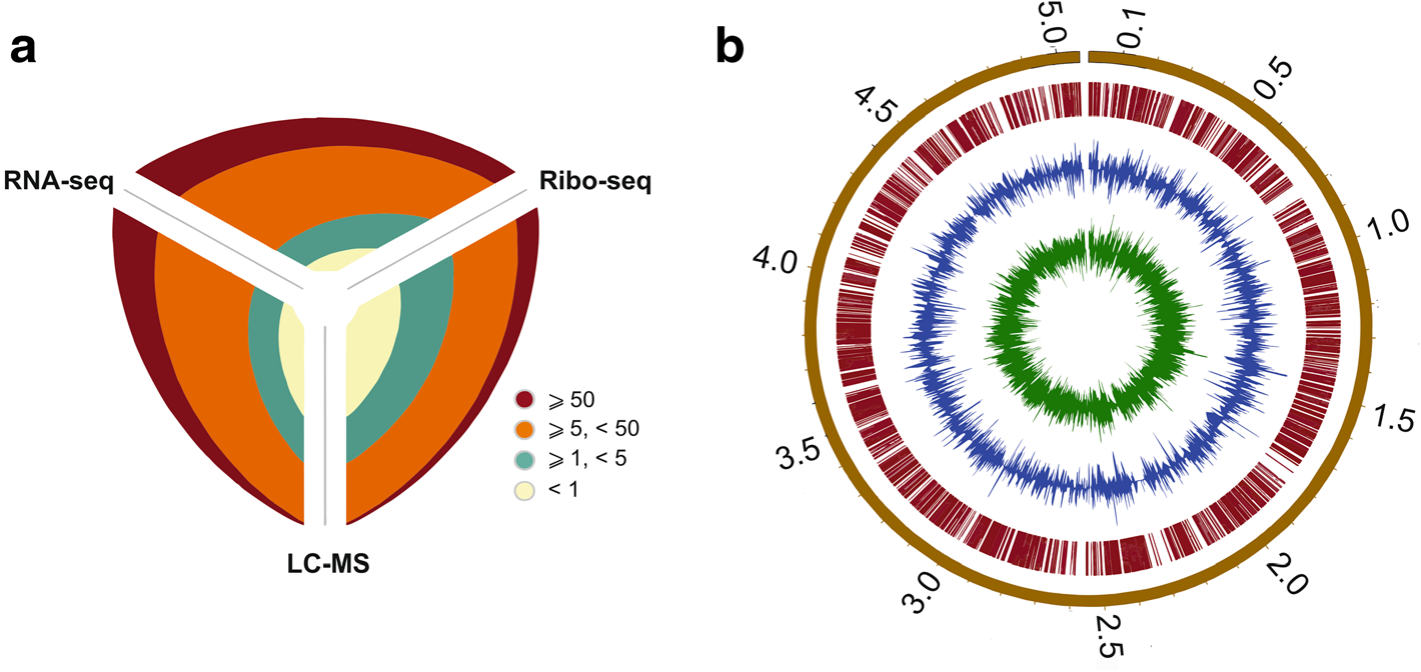
An integrated approach combining RNA-seq/Ribo-seq and Proteomics identifies the coding potential of *M. abscessus*. **a** The breakdown of identified CDSs in MAB using the integrated data map represented as a hive plot. In the inset, expression values are given in color-coded RPKM or peptide counts. **b** Circos image of normalized whole-genome depth of coverage of RNA-seq (green), Ribo-seq (blue) and mass spectrometry (red) data. To aid visualisation, RNA-seq and Ribo-seq expression values are given on logarithmic scale (RPKM values < 1 are assigned a value of 0)

We utilized differential RNA sequencing to determine the primary transcriptome of MAB in logarithmic phase of growth identifying a total of 7,218 transcription start sites (TSS). Analysis of mapped TSS in combination with RNA-seq data facilitated the identification of promoters and regulatory RNA elements within 5′ untranslated regions (5′ UTRs) of annotated genes (Additional file 1: Dataset S1 & Additional file 2: Dataset S2). To identify translated open reading frames (ORFs) we utilized high throughput sequencing of ribosomally protected fragments (Ribo-seq) and LC-MS-based proteomics of trypsin-digested peptides obtained from whole cell lysates. We could identify 4,106 (83 %) of the predicted coding sequences (CDS) using at least one technique while 1,774 (36 %) were present in all of the combined techniques (Fig. 1 & Additional file 2: Dataset S2).

### Integrated profiling indicates the potential for protein isoforms of MAB MviN

**Fig. 2:**
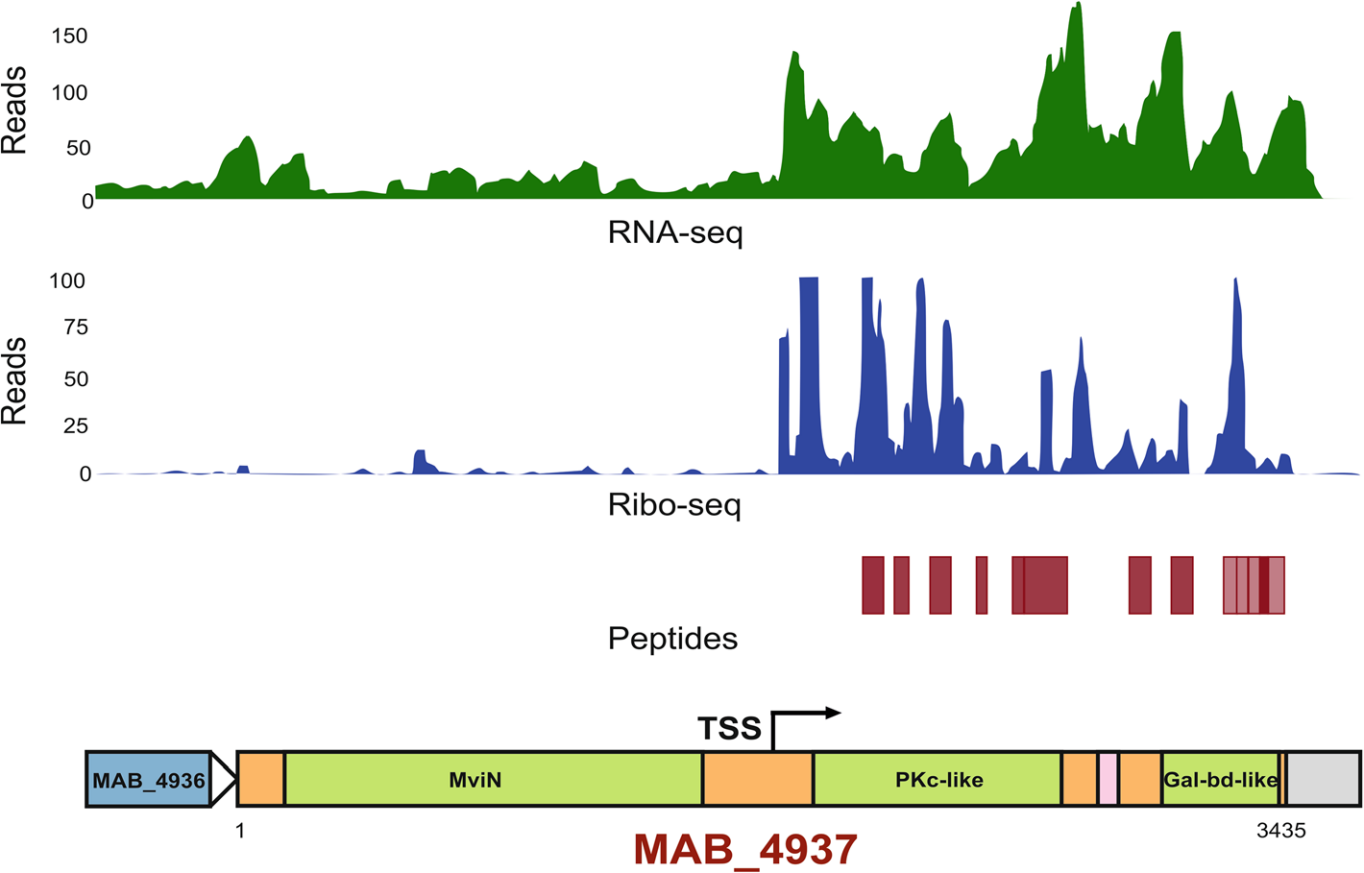
Domain based alternate isoforms of MviN predicted in *M. abscessus* driven by an internal TSS. Presence of isoform is evidenced by a combination of RNA-Seq, Ribo-seq and LC-MS peptide data downstream of the internal TSS

Our integrated map lends support for an internal TSS potentially driving a truncated isoform of the MAB MviN homolog (MAB_4937) (Fig. 2). MviN orthologs in mycobacteria, unlike other MviN proteins, are fused to a C-terminal region containing a predicted intracellular kinase domain and an extracellular sugar binding domain. Transposon mutagenesis studies in *M. tuberculosis* have indicated that the N-terminal region of MviN is essential for in vitro growth while the C-terminal region is dispensable for in vitro growth [3]. The presence of an internal TSS could still facilitate translation of the C-terminal domain of MviN under conditions where the full-length protein is not expressed. Such potential for different protein isoforms originating from internal TSS offers a plausible mechanism for partial essentiality and is illustrative of the potential proteomic diversity open to bacterial genomes.

### Ribosome profiling identifies numerous ribosomally protected short ORFs

Small proteins, generally defined as less than 50 amino acids, tend to be under-predicted by computational screens and present challenges in their biochemical identification primarily due to losses during sample preparation. Ribosome profiling has facilitated the identification of large numbers of small proteins through sequencing of ribosomally protected short ORFs (sORFs). Ribo-seq has also allowed for the identification of leader peptides encoded by upstream ORFs (uORFs) in the 5′UTRs of genes and peptides encoded by sORFs within regulatory RNAs [4]. We identified 126 new ribosomally protected ORFs within the MAB genome 80 % of which are ≤ 50 amino acids in length (Additional file 2: Dataset S2), although this is likely to be an underestimate of the total number of such sORFs within the MAB genome. Thirteen of these new ORFs were restricted to MAB or closely related lineages while the remainder showed varying levels of sequence similarity to homologs from other mycobacterial and related actinomycete genomes. Certain intergenic sORFs despite displaying evolutionary conservation with other mycobacteria showed conflicting indications of their potential to be translated. MAB_4988c and MAB_4957 have numerous mycobacterial homologs including the *M. tuberculosis* (MTB) H37Rv ORFs RVBD_1144Ac and RVBD_3857Ac. However both MTB ORFs have been deleted from subsequent MTB H37Rv genome annotations. RVBD_1144Ac falls between Rv1144 and Rv1145, in a highly transcriptionally active region which has been reported to contain a predicted ncRNA MTS0900 [5]. The ribosomally protected sORFs and significant levels of amino acid conservation despite extensive nucleotide changes suggests however their potential to be translated (Additional file 3: Figure S1). Other intergenic sORFs with annotated homologs in other mycobacterial genomes have homology to intergenic regions (IGR’s) of the MTB H37Rv genome but are absent from the MTB H37Rv annotation. These include, MAB_4957 with homology to IGR (Rv3847c-Rv3848c), MAB_4960c with homology to IGR (Rv3836-Rv3837c), MAB_5021c with homology to IGR (Rv2635-Rv2636).

### Evolutionary conservation of identified upstream ORFs (uORFs)

We identified a number of ribosomally protected sORFs within the leader regions of a number of MAB genes including amino acid biosynthesis operons and metal transporters (Additional file 2: Dataset S2). Although such uORFs can have *trans*-acting roles, typically progression of the ribosome through the uORF varies in response to a physiological signal within the cell, thus influencing the expression of the downstream gene. A number of the uORFs share sequence homology with uORFs previously predicted in the leader regions of their orthologs from *M. smegmatis* and *M. tuberculosis* [6]. These include uORFs upstream of MAB_1662 (*nirA*), MAB_3424c (*leuA*), MAB_3323c (*ilvB*), the transcriptional regulator MAB_4059c, the membrane spanning protein MAB_0741 and a TQXA domain containing protein MAB_0219. We identified uORFs upstream of a number of metal transporters including the magnesium transporters *mgtC & mgtE* (MAB_3593 & MAB_2717c) and the copper-translocating P-type ATPase (MAB_0449). Tandem attenuators have been reported within the 5′UTRs of certain Mg^2+^ transporters allowing their leader regions to be responsive to a number of physiologically diverse signals [7]. *mgtE* (MAB_2717c) appears to contain both a Mg^2+^-responsive *ykoK* leader or M-box co-incident with a ribosomally protected short uORF. The uORF is a homolog of the *vapA* co-expressed virulence gene *vcgB*, restricted to *Rhodococcus equi* and a number of predominantly pathogenic mycobacteria, and contains a downstream binding site for the Mg^2+^-responsive transcriptional regulator PhoP [8]. Although the exact function of VcgB is unknown it is up-regulated upon entry into macrophages and is regulated by increases in temperature and changes in pH levels [9].

### Ribosome protected RNAs and antisense sORFs

One feature of ours and others Ribo-seq data is the presence of ribosomally protected footprints covering parts of the genome which are not obviously protein coding. Ribo-seq in eukaryotes has found that ribosome isolation by sedimentation isolates both ribosomes and Ribonucleoproteins (RNPs) resulting in the ribosomal protection of some prominent classical noncoding RNAs [10]. This phenomenon has not been analyzed in prokaryotes however an inspection of published Ribo-seq data from *E. coli* indicates that the large majority of its characterized ncRNAs display a ribosome footprint (data not shown) [11]. A number of our ribosomally protected fragments encompassed regions overlapping canonical non-coding RNAs such as tRNAs, homologs of annotated mycobacterial small regulatory RNAs (sRNAs) and a number of other ribo-regulators including predicted riboswitches (Additional file 2: Dataset S2). Homologs of conserved mycobacterial sRNAs displaying significant ribosomal reads which are evident both in our data and recently published Ribo-seq data from *M. smegmatis* include homologs of 6C, F6 and MS_IGR-5 in both MAB and in MSMEG, and also MS_IGR-7 (RF02469), MS_IGR-4 (RF02468), MS_IGR-2 (RF02467), Ms_AS-4 (RF02464) and Ms_AS-8 (RF02466) in MSMEG (35). A number of ribosomally protected sORFs were found antisense to MAB proteins including MAB_0366, MAB_1487c, MAB_0590c, MAB_1957, MAB_3309 and MAB_4762.

### Coding potential for ribosomally protected ORFs

**Fig. 3:**
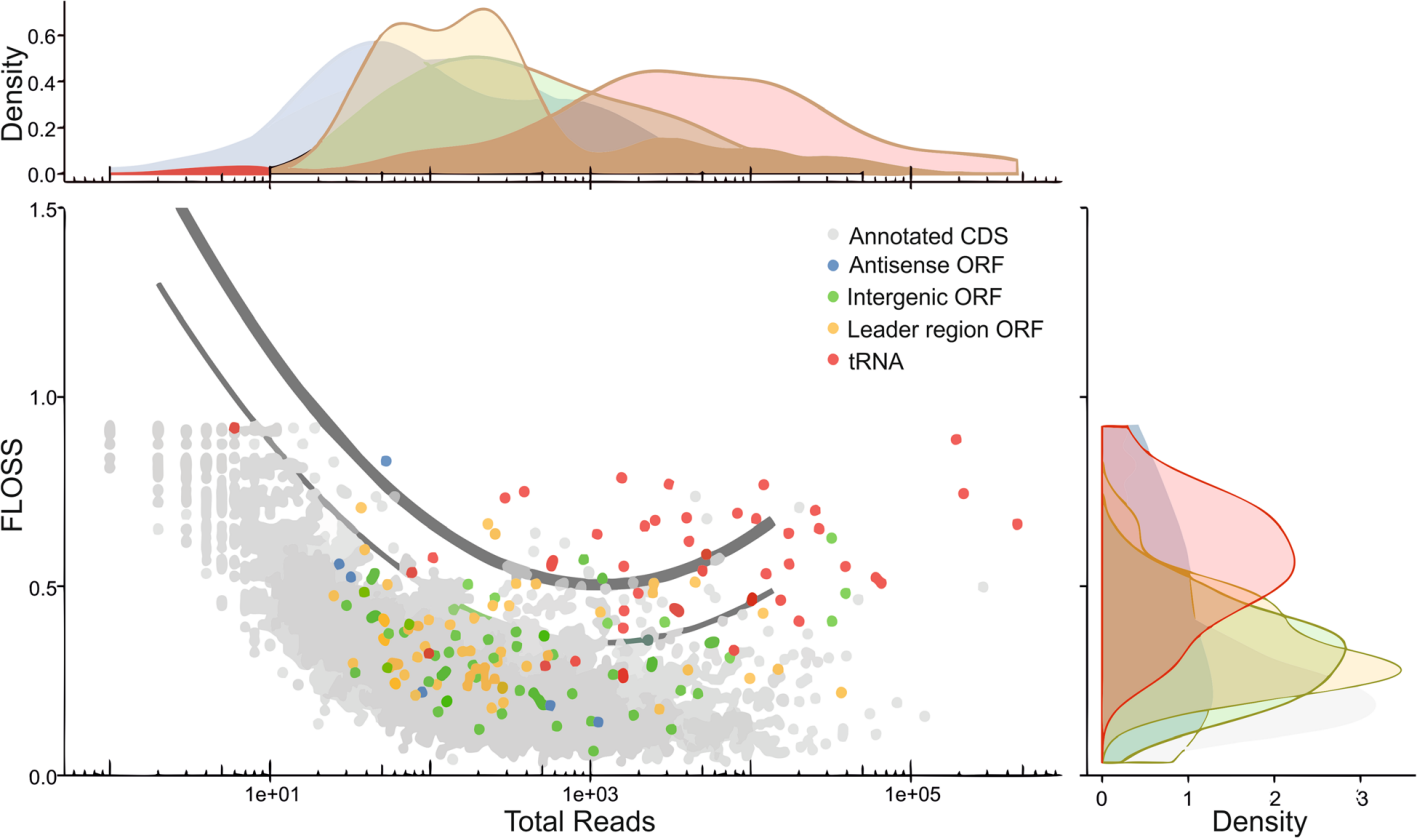
Fragment distribution analysis performed using a fragment length organisation similarity score (FLOSS). With a view to ascertaining the conformity to ribosome profiling read fragment length distributions for coding sequences (CDS), normalised histograms of fragment lengths corresponding to putative coding ORFs and non-coding genes were compared to a reference distribution represented by the mean of the normalised histograms of fragment lengths for all coding features. Curves are representative of outlier thresholds for standard (thinner curve) and extreme (thicker curve) cases using Tukey’s method. Higher FLOSS suggest less conformity. FLOSS are plotted as a function of total reads aligned to each feature. Marginal densities are also given for read counts (x-axis) and FLOSS (y-axis)

The presence of classical non-coding RNAs, antisense sORFs and homologs of known mycobacterial regulatory RNAs in our Ribo-seq dataset raises the question as to whether such sORFs are likely to be coding. This is difficult to clearly assess however in eukaryotic Ribo-seq datasets one method to determine whether long noncoding RNAs (lncRNAs) are likely to be translated is to look at the distribution of the fragment lengths of their ribosomal footprints in comparison with those from known coding ORFs [10]. A fragment length organization similarity score (FLOSS) is then calculated that measures the magnitude of disagreement between the distributions, one corresponding to the feature in question and one corresponding to the mean reference distribution for CDSs, with lower scores reflecting higher similarity. In order to address the coding potential of the predicted sORFs in MAB we calculated FLOSS scores for potentially coding sORFs and compared them with those of the known coding regions within the genome [10] (Fig. 3). In order to contrast with the true ribosome footprints we utilized ribosomal reads covering tRNAs as a canonical non-coding RNA species. Our analysis shows the clear separation of the tRNAs and illustrates that a number of the intergenic and leader sORFs have fragment length distributions indicative of their potential to be translated.

### Transcription profiling of MAB under infection-relevant conditions

Transcriptional responses to nutritional, redox and antibiotic stresses likely underpin the development of inducible antibiotic resistance, persistence and host adaptation in MAB. As few datasets exist we looked at MAB transcriptional response to number of stresses including exposure to antibiotics, nitric oxide (NO) induced hypoxia and growth in an artificial sputum medium designed to mimic the conditions within the Cystic Fibrosis (CF) lung environment [12].

### Characterization of the MAB hypoxic response

Mycobacteria are obligate aerobes with an ability to survive and metabolize under hypoxic conditions. The signature transcriptional response to NO induced hypoxia in MTB is the activation of a dormancy (DosR) regulon, which is important for its long-term persistence within the host [13]. MAB displays cross-reactive immunity to DosR regulon-encoded antigens [14] and is predicted to contain the smallest and minimal DosR regulon consisting of 5 DosR regulated operons [15]. Following NO induced hypoxia using a 40 min pulse of diethylenetriamine/nitric oxide (DETA/NO) (Additional file 4: Document S1), we observed strong up-regulation of the entire MAB predicted DosR regulon including Dos S & R (MAB_3890, MAB_3891), the nitroreductase (MAB_3903), USPs (MAB_2489 & MAB_3904) and an ortholog of Rv2004c (MAB_3902c). In addition the operon MAB_3937-MAB_3939 encoding the Clp ATPase *clpX* and the deazaflavin-dependent nitroreductase (*ddn*) which is important for protection against oxidative stress was also strongly up-regulated (Additional file 5: Dataset S3). Members of the mycobactin cluster *mbtG* and *mbtH* which are up-regulated upon NO exposure in MTB [16] were both up-regulated. A shift in lipid metabolism also appears to be a feature of the hypoxic response in MAB with up-regulation of a number of lipid biosynthesis genes including two homologs of the acyl (ACP) membrane-bound desaturase *desA1* (MAB_3354, MAB_2157).

### Exposure to different antibiotics induces diverse forms of ribosomal stress

MAB responds poorly to antibiotic chemotherapy exhibiting innate resistance to all major anti-tuberculous drugs in addition to a number of broad-spectrum antibiotics. Current regimens often entail administration of both an aminoglycoside antibiotic (AGA) and a macrolide. In response to erythromycin exposure MAB induces an erythromycin resistance methylase gene (*erm41*) [17] which confers resistance. Resistance to AGA is thought to be mediated through a number of aminoglycoside-modifying enzymes (AME), including an aminoglycoside-2-acetyltransferase, Aac(2′) [18]. Under conditions previously described [1, 17] we subjected MAB to a short (1 h) exposure to 1 μM erythromycin or 1 μM kanamycin. Erythromycin exposure resulted in a strong response with 287 genes being differentially expressed (DE) while kanamycin exposure resulted in 73 DE genes (Additional file 5: Dataset S3). The over-represented Clusters of Orthologous Groups (COG categories) in erythromycin-treated cells included Translation, ribosomal structure and biogenesis (category J) (>5-fold enriched; FDR = 6.47E-18) and Amino acid transport and metabolism (category E) (>2-fold enriched; FDR = 4.8E-03). In response to kanamycin, only COG category O (Posttranslational modification, protein turnover, chaperones) was highly enriched (>8.5 fold) (FDR = 9.25E-10) (Additional file 6: Figure S2). Erythromycin exposure induced up-regulation of the 50S and 30S ribosome in addition to numerous tRNA synthases: ArgS (MAB_1433 & MAB_3683c), ValS (MAB_1603), IleS (MAB_2705c), TrpS (MAB_3683c), likely reflecting a response to a rapid decline in protein synthesis. The histidine biosynthesis pathway was also strongly up-regulated in addition to genes associated with homoserine (MAB_0343), asparagine (MAB_1142) and isoleucine (MAB_3321c) biosynthesis. In contrast kanamycin-exposed cell strongly induced genes associated with the heat shock response encompassing the operon MAB_4273c-MAB_4370c (*dnaK, grpE, dnaJ, hspR*), the chaperonins *clpB, groEL*, and *groES*, and their associated regulatory proteins *hspR* and *grpE* (Additional file 5: Dataset S3). Kanamycin exposure also triggered upregulation of the cold shock protein A (MAB_0487) and a predicted alkaline shock protein (MAB_1247c).

### Antibiotic exposure induces multiple antibiotic resistance genes and transporters

Differentially expressed genes from both antibiotic exposures were analysed with ARG-ANNOT [19] to identify homologs of known antibiotic resistance genes. In total, 18 predicted resistance-associated genes were found to be differentially expressed in response to at least one of the two antibiotic treatments (Additional file 7: Table S1). Active efflux mechanisms represent one of the most important aspects of intrinsic antibiotic resistance in mycobacteria [20] and we found a relatively high number of transporters to be differentially expressed upon antibiotic treatment (Additional file 8: Table S2). These included MAB_2640c, a homolog of the multidrug resistance protein (MMR) previously shown to mediate the efflux of several different antibiotics [21] and MMPL13b of the mycobacterial membrane protein large (MmpL) family both induced in response to erythromycin.

### MAB response to artificial CF sputum induces a low energy transcriptional response

Long-term persistence of MAB during chronic infection is a well-established phenomenon however its molecular adaptation to the CF lung environment is poorly understood. As adaptations to the sputum (mucus) layer of the CF lung likely shape clinically relevant traits we determined the transcriptional response of MAB to a brief 3 h exposure to a defined, synthetic CF sputum medium (SCFM2) that mimics the nutritional composition of CF sputum (Additional file 4: Document S1) [12]. Overall, we found the COG category Replication, recombination and repair (category L) to be >3-fold under-represented during growth in artificial sputum (FDR 4.1E-3), while Secondary metabolites biosynthesis, transport and catabolism (category Q) was >1.5-fold enriched (FDR < 0.05) (Additional file 6: Figure S2). Overall this is suggestive of a shift to slow growth, reflected in the down-regulation of the translation apparatus including most ribosomal proteins and several tRNA synthases. Evidence of a clear metabolic shift amongst the topmost up-regulated genes could be clearly identified with parallels to transcriptional changes in a nutrient starvation model of persistence in MTB and growth of MSMEG under low energy conditions [22]. We identified strong induction of the pyruvate dehydrogenase complex (MAB_4918c-MAB_4915c), alongside strong up-regulation of the proton-transporting type-I NADH dehydrogenase (NDH1) operon. NDH1 functions as the primary aerobic respiratory chain and produces ATP via the oxidation of NADH to NAD+ suggesting that energy-limited cells were switching to more energy-efficient mechanisms for membrane potential generation. Under energy-limited conditions MSMEG has been shown to utilize alternative primary dehydrogenases to power its respiratory chain [22]. We identified strong up-regulation of the succinate dehydrogenase (*sdh1*) operon (MAB_4421-MAB_4423) in addition to up-regulation of alanine and proline dehydrogenases.

### Amino acid metabolism and persister genes induced in response to CF sputum

SCFM2 provides amino acids as a major carbon source and studies with the CF pathogen *Pseudomonas aeruginosa* have highlighted the induction of branched chain and aromatic amino acid catabolism genes [23]. *P. aeruginosa* preferentially catabolizes alanine, arginine and glutamate in CF sputum and up-regulation of the anaerobic arginine-deiminase pathway in *P. aeruginosa* is thought to contribute to anaerobic CF lung adaptation [24]. Numerous amino acid metabolism genes including those associated with arginine, lysine and proline catabolism were up-regulated in MAB response to SCFM2 exposure. Mycobacteria have both anaerobic and aerobic pathways for arginine metabolism and SCFM2 exposure caused strong induction of genes associated with the aerobic arginase pathway that facilitates the conversion of arginine to glutamate. These included *rocA*, the operon MAB_3840-MAB_3843 encompassing the ornithine aminotransferase (*rocD*), the arginine permease *rocE* and MAB_3840, a homolog of Rv2323c which is essential for growth on arginine. MTB uses arginine to produce proline and genes associated with proline metabolism are strongly expressed when mycobacterial cells are exposed to nutrient starvation [25]. We observed strong up-regulation of the operon MAB_1330-MAB_1331 which contains pyrroline-5-carboxylate dehydrogenase (*pruA*) and proline dehydrogenase (*pruB*), which oxidizes proline to glutamate for use as a carbon and nitrogen source. One of the most highly up-regulated operons upon SCFM2 exposure MAB_3647c-MAB_3646c contains the alarmone lysine ε-aminotransferase (LAT), downstream of the transcriptional regulator leucine-responsive regulatory protein (*lrpA*). LAT is involved in the conversion of lysine to glutamate and is associated with persister cell formation following nutrient starvation [26]. We also found strong up-regulation of L-alanine dehydrogenase (*MtAlaDH*) (MAB_3100), which is also highly up-regulated under nutrient starvation. We saw up-regulation of the operon MAB_3521c-MAB_3520c containing *nirD* which is important during in vitro dormancy of MTB [27] downstream of the nitrogen responsive transcription factor *nnaR*.

### Fatty acid catabolism genes induced in response to CF sputum exposure

*P. aeruginosa* is known to up-regulate fatty acid catabolism genes in SCFM [28] and we observed strong up-regulation of members of the fatty acid β-oxidation pathway including *fadD* and *fadE*. The acyl-CoA carboxylase alpha and beta subunits (*accA & accD*) were also strongly up-regulated suggestive of increased levels of propionyl-CoA as a result of the degradation of odd-chain fatty acids or branched-chain amino acids. In MTB, propionyl-CoA generated from metabolism of host lipids is used as a substrate to generate cell wall lipids which represents an important adaptation and is essential for survival within the host [29]. We identified strong down-regulation of the mycolate pathway including downregulation of the β-ketoacyl-ACP synthases (*kasA & kasB*) and beta-ketoacyl synthases MAB_2031& MAB_2029 and the malonyl-CoA:acyl carrier protein transacylase (MCAT) homolog (MAB_2034). Down-regulation or deficiency in *kasA/B* has been associated with altered colony morphology in MTB [30] and in MSMEG, in which the transition to a ‘biofilm phase’ involves reduction in levels of the KasA protein [31].

### Down regulation of iron acquisition and siderophore production genes

A number of virulence factors of *P. aeruginosa* are switched off during progressive CF lung infection including genes associated with siderophore production [32]. With the exception of *mbtH* we observed strong down-regulation of the mycobactin cluster encoding the biosynthetic enzymes for assembly of the siderophore mycobactin. We observed strong up-regulation of the ESX-3 cluster (MAB_0149c-MAB_0147c) containing the pair of *pe*/*ppe* genes (*pe5* and *ppe4*) and the EspG family protein. The ESX-3 locus is proposed to be involved with acquisition of divalent metal ions however it is also proposed to play other roles in virulence [33].

### Transcriptional repressors predominate in response to CF sputum growth

We identified the transcriptional regulator *lsr2* as one of the topmost induced genes upon exposure to SCFM2. Lsr2 works mainly to repress gene transcription through binding AT-rich sequences in MTB and is critical for persistence and a shift from aerobic to anaerobic respiration [34]. We also observed strong up-regulation of a majority of TetR family transcriptional regulators consistent with an overall transcriptional repression associated with slow growth. In a nutrient starvation model of persistence in MTB sigma factors (*sigB*, *sigE*, *sigF* and *sigD*) are significantly up-regulated while the *rpoA* subunit of RNA polymerase is down-regulated [25]. Exposure to SCFM2 induced a strong up-regulation of the sigma factor *sigB* (MAB_3028), *sigE* (MAB_1362) and *sigH* (MAB_3543c) while *rpoA* was down-regulated. In addition there was a strong up-regulation of a cluster of genes encoding *sigF* (MAB_2511), its anti-sigma regulator *usfX* (MAB_2513c) and MAB_2512, a homolog of the *sigF* regulator (Rv0516c).

### Dissection of MAB transcriptional programmes driving niche adaptation

**Fig. 4:**
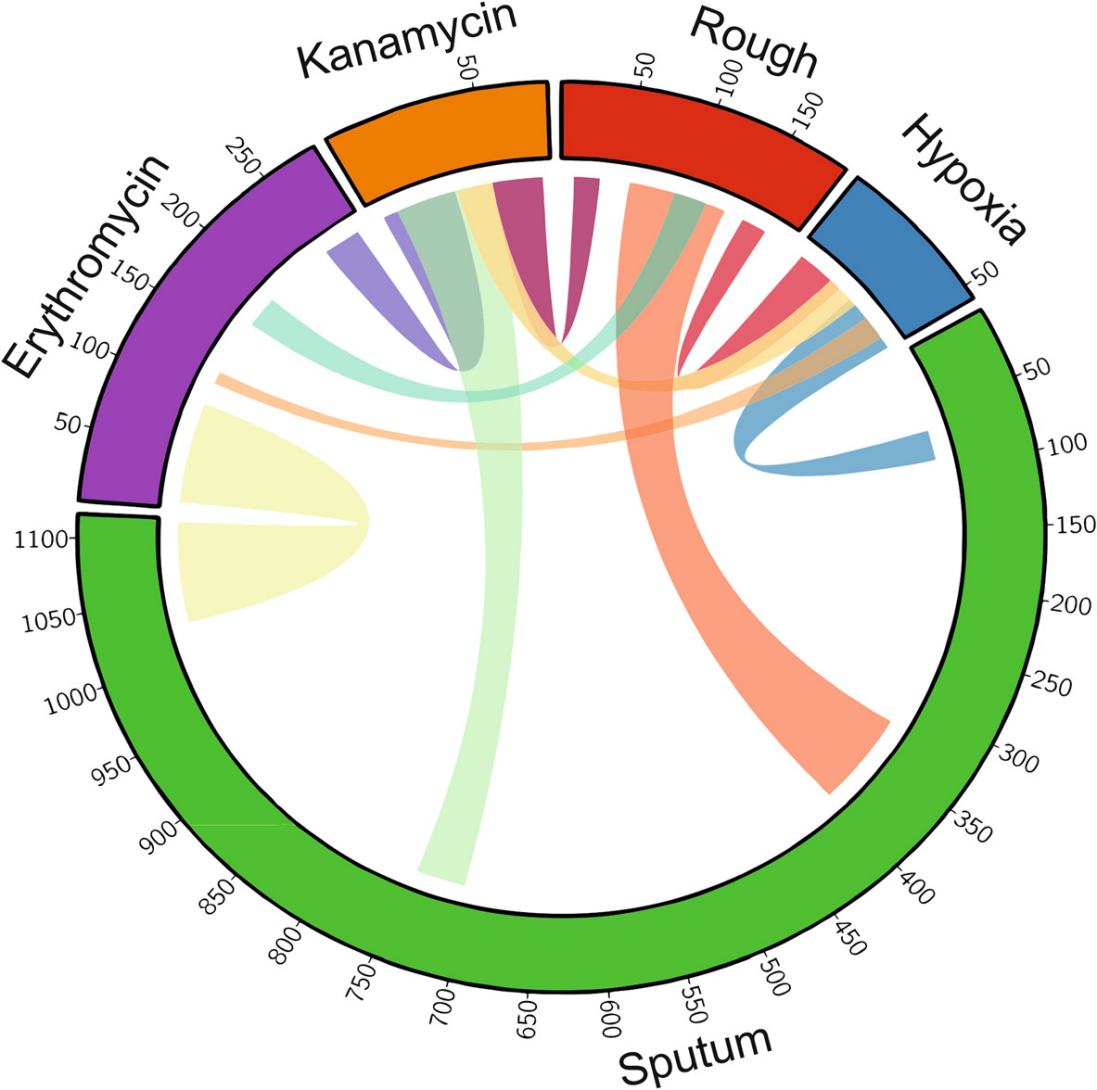
Circos plot illustrating extent of common differentially expressed genes between all tested conditions. The width of each connecting ribbon is directly proportional to the number of shared genes between conditions on either end of the ribbon; the proportion of the circumference of the circle covered by a given treatment condition is directly proportional to the number of differentially expressed genes for that condition

Successful colonization of its human host by MAB is likely to be due to a series of individual niche adaptations. Although the current data is relatively sparse we looked to see whether we could begin deconstructing more complex transcriptional adaptations based upon the sharing of co-expressed genes with more discreet responses. We also compared our datasets to the only other published dataset that associated with the transition from a smooth to a rough morphology (Fig. 4) [35].

Our comparisons reveal some significant overlaps and number of interesting sub-networks that span differing conditions. We find 43 % (22 of 51) of the hypoxic response including the entire DosR regulon is up-regulated in MAB strains with a Rough morphotype, a link that is pronounced in clinical strains with the potential for persistence [35]. Constitutive up-regulation of the DosR regulon in the W-Beijing Lineage of *M. tuberculosis* is thought to confer an adaptive advantage for growth in microaerophilic or anaerobic environments [36]. In contrast, although the hypoxic response and sputum shares a greater overlap (49 % 25/51) only *devR & devS* from the DosR regulon are up-regulated indicating short exposure to SCFM2 does not induce the hypoxic response and that *devR & devS* may be induced for reasons other than hypoxia. This is in keeping with our findings that points towards a metabolic adaptation to a restriction in the diversity of carbon sources upon SCFM2 exposure but missing a clear signature of anaerobic growth. Both antibiotic exposures included induction of the transcriptional regulator *whiB7* (MAB_3508c) alongside the alleviation of a number of TetR transcriptional repressors (MAB_4647/MAB_4687/MAB_2957c). In MTB WhiB7 is induced by both antibiotics and acts in conjunction with the primary sigma factor SigA to activate a discreet regulon of 12 genes controlling intrinsic antibiotic resistance [37]. MAB has 8 homologs of the MTB WhiB7 regulon including *erm41* (MAB_2297), a homolog of the multidrug efflux pump *tap* (MAB_1409c) and a putative macrolide transporter (MAB_1846). In addition to *whib7* up-regulation, erythromycin exposure induced a large majority (7/8) of the MAB homologs of the WhiB7 regulon indicative of an evolutionarily conserved WhiB7 driven response to macrolides. *whiB7* in MAB is also induced in response to sub-inhibitory concentrations of the aminoglycoside amikacin [38] and is the second most strongly induced gene in response to kanamycin exposure. Intriguingly however, in response to kanamycin exposure only two of the 8 MAB homologs of the Whib7 regulon, *whib7* and the putative macrolide transporter MAB_1846 were induced. This uncoupling of WhiB7 induction from upregulation of its predicted regulon was also evident in response to SCFM2 where, despite the large number of differentially expressed genes and a strong induction of *whiB7,* MAB homologs of its regulon were largely unaffected. One of the strongest induced operons in response to both antibiotics MAB_0357c & MAB_0356c encodes a delta-aminolevulinic acid dehydratase (ALAD) and a S-isoprenylcysteine methyltransferase (*mddA*), respectively. MddA has been shown to catalyze production of the volatile compound dimethyl sulphide (DMS) which has been used as a volatile biomarker for differentiating between the growth of different mycobacterial species [39].

### Expression of novel sORFs across different conditions

**Fig. 5:**
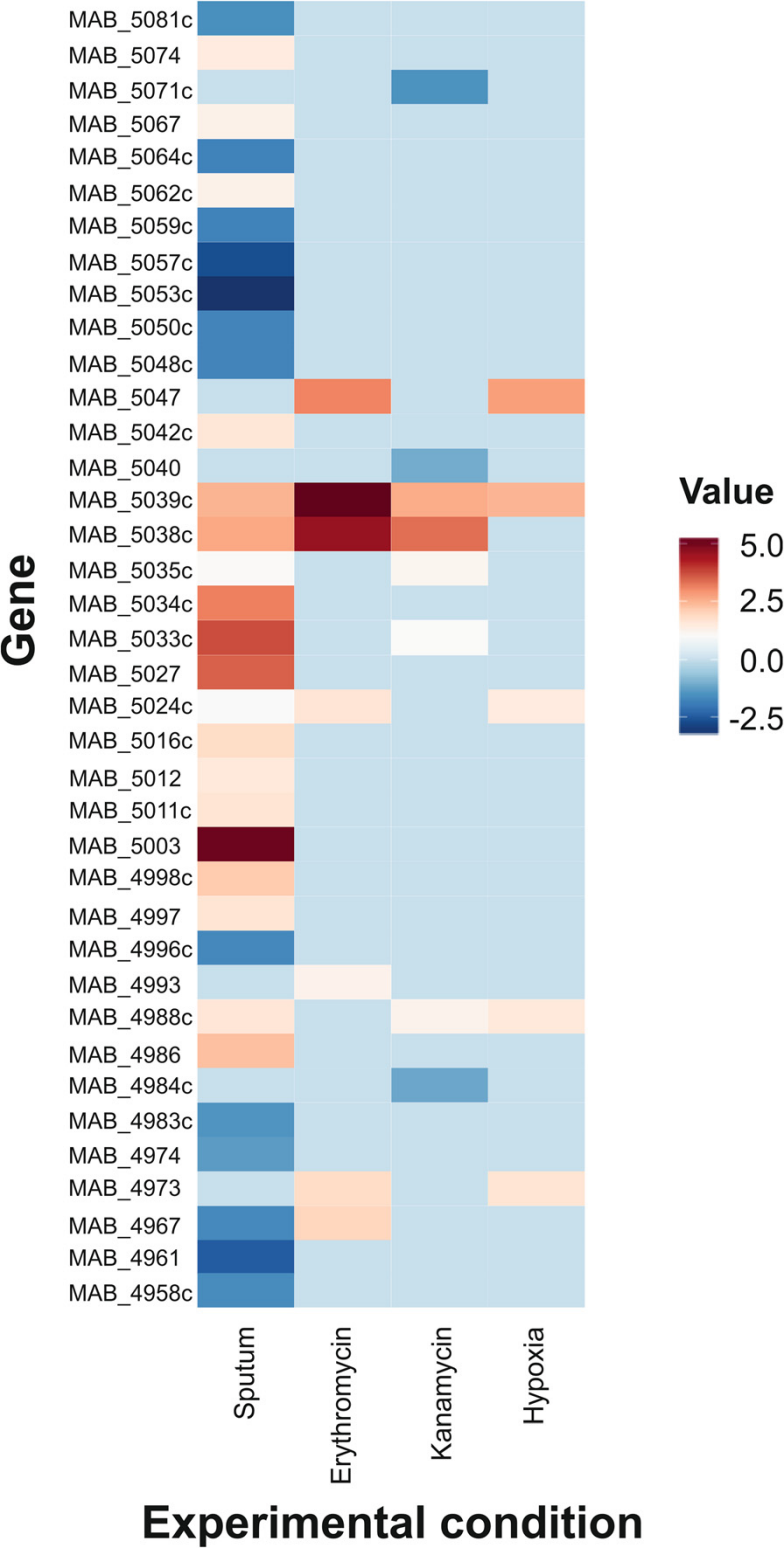
Heat map of gene expression values for novel ORFs in following tested conditions: growth in an artificial sputum, exposure to erythromycin or kanamycin, hypoxia. The color bar corresponds to a range of gene expression values from -2.5 to 5

We looked at expression patterns for the novel sORFs identified by ribosomal profiling across the different conditions (Fig. 5 & Additional file 5: Dataset S3). Their expression patterns overall mirrored those of the remainder of the genome with the majority being down-regulated in response to SCFM2 while much smaller numbers were mostly up-regulated in response to the remaining conditions. Highly up-regulated sORFs (MAB_5038c & MAB_5039c) are in proximity to *whiB7,* which is up-regulated in response to all conditions tested except hypoxia.

## Conclusions

Central to understanding the functional networks that contribute to adaptation and persistence in MAB is a well-characterized genome coupled with rich genome-wide functional genomics datasets. It’s clear from our data that the complexity of the MAB genome is only modestly represented by current automated annotations of its coding regions. We demonstrate the presence of many novel sORFs with coding and regulatory potential and identify internal TSS as a potential source of protein isoforms. Our Ribo-seq data illustrates that many non-coding RNAs, including regulatory and antisense RNAs, are ribosomally protected. As they lack clear evidence of translation their presence means that these antisense RNAs are competing kinetically with the ribosome for access to the same region of the nascent target RNA. Functional regulatory RNAs are difficult to detect in mycobacteria due to the pervasiveness of transcription initiation [40] and identification of RNAs that are ribosomally protected may provide better evidence of their being functional than TSS or RNA-seq alone. We also looked to expand the amount of condition specific transcriptomics data, as despite individual gene association studies, there is almost no genome-wide functional genomics data available for MAB. Such data are necessary to refine and progress beyond a reliance on interpreting MAB biology through the prism of shared orthologs with mycobacteria such as MTB, which has extensive systems-wide datasets (www.tbdb.org). Therefore we profiled MAB response to a number of biologically relevant exposures that have either been experimentally characterized or for which some comparable data is available from other species [12, 16, 22]. In doing so we characterize the *M. abscessus* hypoxic response and the role of the evolutionarily conserved DosR regulon therein. Our data lends greater weight to DosR regulon’s constitutive up-regulation in strains of the Rough morphotype as it may confer an adaptive advantage for survival in the hypoxic CF lung environment. We identify for the first time numerous antibiotic resistance genes up-regulated in response to erythromycin and kanamycin exposure including antibiotic modification enzymes, efflux pumps and the redox sensitive WhiB7 transcriptional regulator. Unexpectedly we find that although *whiB7* and its predicted regulon in MAB are both induced in response to erythromycin this link appears clearly uncoupled in response to other antibiotics and stresses. Given the diversity of WhiB7 activators and its important role in inducible antibiotic resistance this deserves further study. Comparison with transcriptional data from other CF pathogens clearly indicates a convergence in metabolic strategies towards adaption to the CF lung [12, 23, 32]. Our analysis also shows that the response to SCFM2 triggers the activation of metabolic pathways that are required in MTB for growth in human macrophages and pave the way for the development of persistence [22]. Our analysis therefore offers insights into how MAB may transcriptionally adapt long-term to the CF lung and the nutrient limited conditions that prevail within human macrophages. Given what little is known about MAB’s strategies for adaptation and survival, such knowledge is essential for developing new strategies to treat it.

## Methods

Detailed experimental procedures are provided in Additional file 4: Document S1 in the supplemental material.

As the work was carried out on bacterial cultures the authors state that no ethical approval was required for any aspect of this study.

### Bacterial culture conditions

*M. abscessus* ATCC19977 cultures were grown in Middlebrook 7H9 medium (supplemented with 10 % albumin-dextrose-catalase solution, 0.5 % glycerol and 0.05 % Tween-80). When indicated, diethylenetriamine/nitric oxide (DETA/NO) adduct (final concentration of 50 μM), kanamycin or erythromycin (final concentration of 1 μM) were added to the culture media. For growth in an enhanced artificial sputum medium (SCFM2) bacterial cultures were washed with phosphate buffered saline solution (PBS) and then transferred to SCFM2 medium.

### RNA isolation and preparation of samples for high throughput sequencing

RNA was isolated as described earlier using Qiagen miRNeasy kit with double DNase treatment [35]. Sample preparation for dRNAseq and TSS sequencing was carried out by Vertis Biotechnologie AG [40]. For RNA-seq, ribosomal RNA was removed using RiboZero (Epicentre), cDNA libraries were generated with the TruSeq Stranded RNA kit and validated according to manufacturer’s instructions (Illumina Inc.). For ribosome profiling, translation was arrested with 100 mg/mL chloramphenicol, bacteria were recovered, suspended in lysis buffer (100 mg/mL chloramphenicol, 70 mM KCl, 10 mM MgCl2, 10 mM Tris-HCl [pH 7.4]) and physically disrupted. The clarified lysates were digested with MNase and RNaseI. Ribosome protected fragments were prepared for high throughput sequencing according to a protocol reported elsewhere [41]. Samples were sequenced on either a MiSeq System (Illumina Inc.) with a 150 bp single end read mode or a HiSeq 2500 in a 50 bp single read format.

### Reads mapping

Following removal of sequencing adapters and quality control, all ribosome profiling and RNA-seq reads were mapped to the *Mycobacterium abscessus* ATCC 19977 genome sequence (RS: NC_010397, GB: CU458896) using Bowtie 1.1.1. Ribosome profiling and RNA-seq datasets were deposited into NCBI’s Gene Expression Omnibus (GEO) (accession numbers: GSE78787 and GSE72996).

### Whole proteome analysis and tryptic peptide mapping

Recovered bacterial pellets were washed with ice-cold PBS, suspended in protein lysis buffer (100 mM Tris-HCl pH 7.8, 50 mM KCl, 0.5 mM DTT and 6 M urea) and physically disrupted. Clarified total cell lysate was reduced with dithiothreitol (DTT), alkylated with iodoacetamide and subjected to overnight trypsin digestion (Pierce Biotechnology Inc.). Resulting tryptic peptides were desalted by solid phase extraction with C18 ZipTips (Millipore Corporation) and analysed on a Thermo Scientic Q Exactive mass spectrometer connected to a Dionex Ultimate 3000 (RSLCnano) chromatography system.

Tryptic peptides were aligned to the *Mycobacterium abscessus* ATCC 19977 genome sequence (RS: NC_010397, GB: CU458896) using a database search approach implemented in PEAKS 6.0 proteomics software. A parent mass error tolerance of 6.0 ppm was used in conjunction with a maximum of 2 missed cleavages along with one non-specific cleavage.

## Abbreviations

DE, differentially expressed; MAB, *Mycobacterium abscessus*; SCFM, synthetic cystic fibrosis sputum; TSS, transcriptional start site

## Additional files

Additional file 1: Dataset S1. Full list of experimental TSSs identified in *M. abscessus*, with motifs identified within their promoter regions. (XLSX 753 kb) Additional file 2: Dataset S2. List of all annotated genomic features and newly identified elements together with their mapped TSSs and expression levels (transcriptome, translatome and proteome). (XLSX 713 kb) Additional file 3: Figure S1. Nucleotide and amino acid sequence alignments of MAB_4988c (*M. abscessus*), MAP_2636 (*M. avium*), MMAR_4306 (*M. marinum*) and RVBD_1144Ac (*M. tuberculosis* H37Rv). (DOCX 874 kb) Additional file 4: Document S1. Detailed experimental procedures. (DOCX 30 kb) Additional file 5: Dataset S3. List of differentially expressed genes (including new CDS) in four experimental conditions: hypoxia, exposure to erythromycin, exposure to kanamycin and growth in artificial sputum medium. (XLSX 99 kb) Additional file 6: Figure S2. Enrichment of functional categories among differentially expressed genes in response to antibiotic treatment. (DOCX 80 kb) Additional file 7: Table S1. List of genes with predicted roles in mediating antibiotic resistance which are differentially expressed upon antibiotic exposure. (DOCX 12 kb) Additional file 8: Table S2. List of genes encoding predicted membrane transporters which are differentially expressed upon antibiotic exposure. (DOCX 13 kb)
