# Supplementary material for: Functional characterization of the Mycobacterium abscessus genome coupled with condition specific transcriptomics reveals conserved molecular strategies for host adaptation and persistence
Source: BMC Genomics. 2016 Aug 5;17:553. doi: 10.1186/s12864-016-2868-y (PMC4974804; doi:10.1186/s12864-016-2868-y)
Supplement: Additional file 3: Figure S1. — Nucleotide and amino acid sequence alignments of MAB_4988c (M. abscessus), MAP_2636 (M. avium), MMAR_4306 (M. marinum) and RVBD_1144Ac (M. tuberculosis H37Rv). (DOCX 874 kb) [file 12864_2016_2868_MOESM3_ESM.docx]

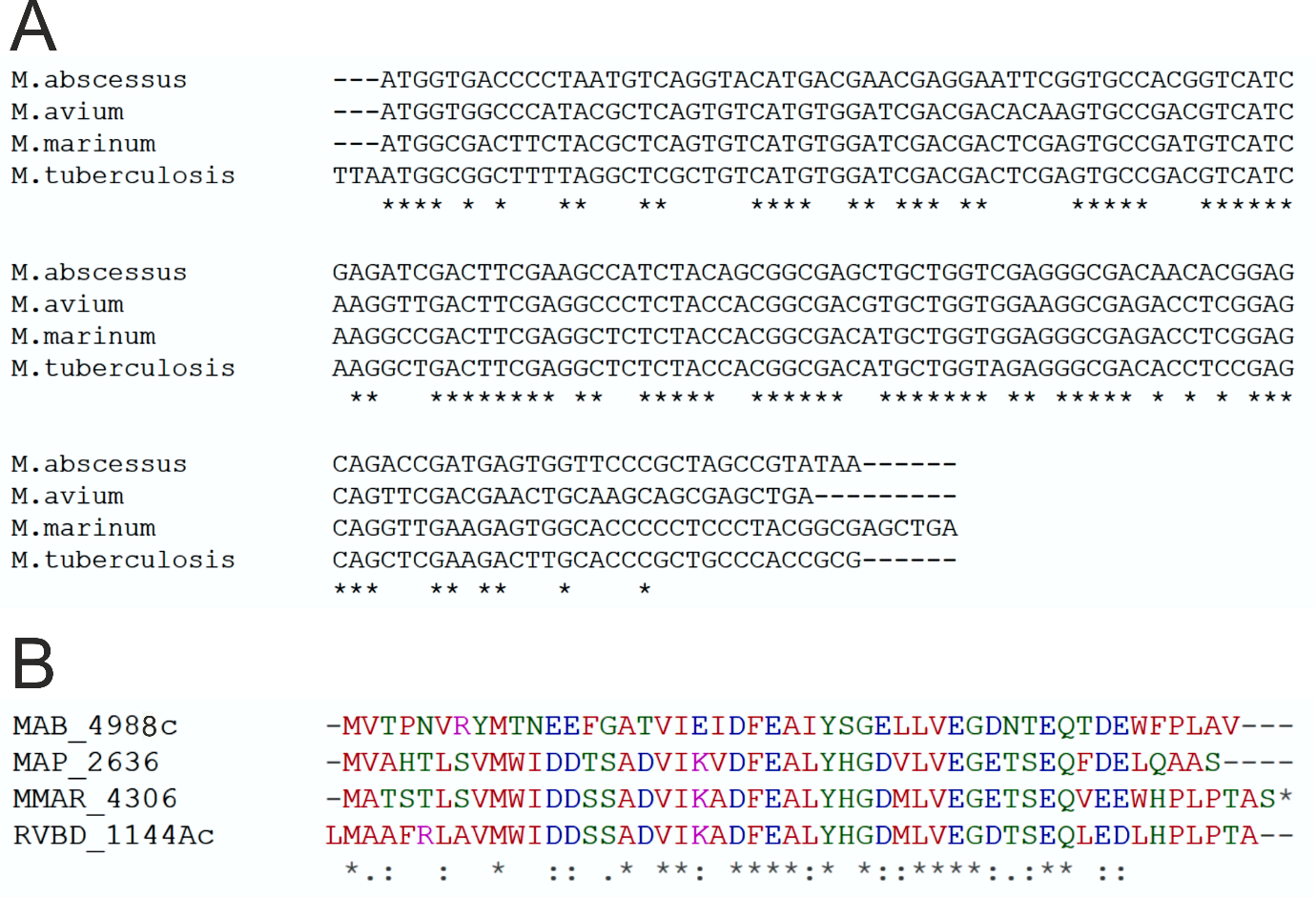


**Additional file 3: Figure S1.** Alignment of A) nucleotide sequences and B) amino acid sequences of *M. abscessus* MAB_4988c identified in this study and corresponding sequences of MAP_2636 (*M. avium*), MMAR_4306 (*M. marinum*) and RVBD_1144Ac (*M. tuberculosis* H37Rv). Sequence conservation is given below each alignment.
