# Supplementary material for: Functional characterization of the Mycobacterium abscessus genome coupled with condition specific transcriptomics reveals conserved molecular strategies for host adaptation and persistence
Source: BMC Genomics. 2016 Aug 5;17:553. doi: 10.1186/s12864-016-2868-y (PMC4974804; doi:10.1186/s12864-016-2868-y)
Supplement: Additional file 4: Document S1. — Detailed experimental procedures. (DOCX 30 kb) [file 12864_2016_2868_MOESM4_ESM.docx]

**Additional file 4**

**Supplemental Experimental Procedures**

**Bacterial culture conditions**

For each set of experiments triplicate *M. abscessus* ATCC19977 cultures were grown in Middlebrook 7H9 medium (supplemented with 10% albumin-dextrose-catalase solution, 0.5% glycerol and 0.05 % Tween-80) with continuous shaking at 37 ᵒC. To construct a multi-layered expression map bacterial cultures were grown to OD600 = 0.8, then split into three aliquots and subsequently prepared for RNAseq, ribosome profiling and whole proteome analyses.

To induce hypoxia, a 40 min pulse of diethylenetriamine/nitric oxide (DETA/NO) adduct was applied (final concentration of 50 μM) [1]. For antibiotics treatment, kanamycin or erythromycin was added to the culture media to a final sub-inhibitory concentration of 1 µM and cultures were propagated for 1 h [2,3]. To test bacterial response to conditions resembling environment of a cystic fibrosis lung, following 3 washes in phosphate buffered saline (PBS), bacteria were transferred to an enhanced artificial sputum medium [4] and incubated for 3 h. All experiments were carried out using triplicate cultures and were compared to not-treated cultures grown in standard Middlebrook 7H9 medium.

**RNA isolation and RNAseq sample preparation**

Bacteria recovered by centrifugation were treated with Trizol (Sigma Aldrich) and physically disrupted with 0.1 mm zirconia-silica beads using MagNA Lyzer (2 pulses, 30 s each) (Roche Diagnostics Ltd.). Following chlorophorm/isopropanol precipitation, DNA was removed by 30 min digestion with Turbo DNase (Ambion). RNA was isolated using miRNeasy spin columns with an additional on-column DNase treatment, according to manufacturer’s instructions (Qiagen). RNA was quantified using NanoDrop 1000 spectrophotometer (Thermo Scientific) and sample quality was assessed using RNA Nano Chip on a Bioanalyzer 2100 instrument. Total RNA was treated with RiboZero (Epicentre) to deplete ribosomal RNA. Sequencing libraries were generated with the TruSeq Stranded RNA kit according to manufacturer’s instructions (Illumina Inc.). High Sensitivity DNA chip on Bioanalyzer 2100 (Agilent) was used to determine library quality, while the concentration was determined using Qubit dsDNA HS assay (Thermo Fisher). For the multi-layered map cDNA libraries were sequenced on the MiSeq System (Illumina Inc.) with a 150 bp single end read mode in house. For the transcription profiling of bacteria exposed to stress conditions samples were sequenced on a HiSeq 2500 by Michigan State University in a 50 bp single read format.

**dRNAseq**

Sample preparation and TSS sequencing was carried out by Vertis Biotechnologie AG as previously reported [5]. Briefly, prior to library construction, half of each RNA sample was treated with Terminator 5'-Phosphate-Dependent Exonuclease (TEX), an enzyme which specifically degrades processed RNA transcripts carrying a single 5' phosphate group, leaving only primary transcripts carrying a tri-phosphate at the 5' end. The other half of each RNA sample was left untreated, and hence was not enriched for primary transcripts. All RNA was then converted to cDNA for library preparation and sequenced on an Illumina Genome Analyser IIx (GA IIx).

**Ribosome profiling**

To arrest translation bacterial cultures were treated with 100 mg/mL chloramphenicol and then spun down (10 min at 4000 x g). The bacterial pellets were suspended in lysis buffer (100 mg/mL chloramphenicol, 70 mM KCl, 10 mM MgCl2, 10 mM Tris-HCl [pH 7.4]) and physically disrupted by two passages through a French Press (10,000 psi), followed by centrifugation to remove intact cells and cellular debris. The clarified lysates were digested with MNase and RNaseI. Ribosome protected fragments were recovered by sedimentation through a 1 M sucrose cushion, size selected by gel purification and prepared for high throughput sequencing [6]. Briefly, 3’ termini of ribosome protected fragments were dephosphorylated with polynucleotide kinase (PNK, New England Biolabs) allowing for ligation of a 3’ RNA TruSeq small RNA adapter (Illumina Inc.). The 5’ termini were then phosphorylated by PNK and 5’ RNA TruSeq small RNA adapter was ligated. Following reverse transcription, the ribosome protected fragments were amplified by PCR and PCR products of expected size were purified from a Tris-Borate-EDTA gel. After quality control on Bioanalyzer 2100 and library quantification, samples were sequenced on the HiSeq 2500 System in a 50 bp single end read mode.

**Whole proteome analysis**

Bacterial pellets were recovered by 10 min centrifugation at 4000 x g. Following three washes with ice-cold PBS, samples were resuspended in protein lysis buffer (100 mM Tris-HCl pH 7.8, 50 mM KCl, 0.5 mM DTT and 6 M urea) and physically disrupted using 0.1 mm zirconia-silica beads in MagNA Lyzer (Roche Diagnostics). After a brief centrifugation total cell lysates were collected and protein concentration was determined using DC Protein Assay (BioRad). 1 mg of total protein was reduced with dithiothreitol (DTT) and alkylated with iodoacetamide, followed by removal of unreacted alkylating reagent with DTT. Diluted samples were subjected to overnight trypsin digestion (Pierce Biotechnology Inc.). The reactions were stopped by decreasing the pH to < 6 with concentrated acetic acid. Tryptic peptides from 10 µl aliquots of the digests were desalted by solid phase extraction with C18 ZipTips (Millipore Corporation) and eluted in 50% acetonitrile.

The samples were run on a Thermo Scientic Q Exactive mass spectrometer connected to a Dionex Ultimate 3000 (RSLCnano) chromatography system. Tryptic peptides were resuspended in 0.1% formic acid. Each sample was loaded onto a fused silica emitter (75 m ID, pulled using a laser puller (Sutter Instruments P2000)), packed with Reprocil Pur C18 (1.9 µm) reverse phase media and was separated by an increasing acetonitrile gradient over 90 minutes at a low rate of 250 nl/min. The mass spectrometer was operated in positive ion mode with a capillary temperature of 320 ᵒC, and with a potential of 2300V applied to the frit. All data was acquired with the mass spectrometer operating in automatic data dependent switching mode. A high resolution (70,000) MS scan (300-1600 m/z) was performed using the Q Exactive to select the 12 most intense ions prior to MS/MS analysis using HCD.

**Transcription Start Site (TSS) mapping**

TSS locations were identified by using a custom Perl script to compare the 5' read depths of enriched primary transcripts (TEX+ library) relative to unenriched total transcripts (TEX- library). The coordinates of the 5' ends of all uniquely mapped TEX+ library reads were determined, and the TEX+/TEX- read ratios were calculated at each position in the genome. Sites with a significant enrichment of TEX+ to TEX- reads (i.e. an enrichment of primary transcripts) were considered candidate TSSs. These were then annotated, using custom Python scripts, according to their locations relative to annotated genes.

**Reads mapping**

All ribosome profiling and RNA-seq reads were trimmed for Illumina Truseq adapters and subsequently mapped to the *Mycobacterium abscessus* ATCC 19977 genome sequence (RS: NC_010397, GB: CU458896) using Bowtie 1.1.1 [7]. The sequencing data described in this report have been submitted to the NCBI gene expression omnibus (GEO) under accession numbers GSE78787 and GSE72996.

**Differential gene expression**

A custom annotation file, containing annotated and novel coding regions, as well as known mycobacterial sRNAs, was compiled, and read counts per gene were obtained using a custom Python script in combination with SAMtools [8]. The EdgeR Bioconductor package [9] was then used to perform estimation of gene-wise dispersion and differential expression analysis using the exact test. Multiple testing correction was implemented using the Benjamini-Hochberg approach.

**Clusters of Orthologous Groups (COG) enrichment analysis**

Annotation of M. abscessus genes according to COG category was performed by sequence homology using the WebMGA database [10]. The numbers of DE and non-DE genes in each category were then calculated, and enrichment analysis for each category was carried out using the chi-squared test of independence of variables in Python. Multiple testing correction was implemented in R following the method of Benjamini and Hochberg.

**CDS mapping**

Both ribosome profiling read density and the LC-MS-derived tryptic peptides were mapped to the *Mycobacterium abscessus* ATCC 19977 genome sequence. Using the most up-to-date GenBank annotation (accession number NC_010397) as a reference, an RNA-seq score >= 5 RPKM, a ribosome profiling score >= 5 RPKM and/or the discovery of at least one tryptic peptide found to overlap the coordinates of the CDS was required to identify a given CDS. In order to perform this analysis, the R programming language (3.0.2) [11], the Python programming language (2.7.11) [12], SAMtools (0.1.19-44428cd) [8] and BEDtools (v2.22.0) [13] were extensively used.

**Identification of Putative ORFs**

In order to identify putative coding ORFs, an iterative pipeline was implemented. Specifically, an original compendium of candidate ORFs was generated comprising all intergenic nucleotide sequences beginning with an ATG/GTG codon, terminating at a downstream in-frame stop codon and containing non-zero ribosome profiling footprints. These candidates were then scored using a read-fit measure: specifically, for a given candidate, this was computed by calculating the product of a scaling factor, representing the number of above-background read expression coordinate positions, and the sum of the raw read counts at each coordinate position belonging to the feature's open reading frame. The ability to assign a rank to each putative ORF based on this measure enabled the principled elimination of sub-ORFs as well as candidate ORFs with prohibitively large overlap with other features. In addition, a combination of extra feature analysis such as length, genomic conservation, genomic neighbourhood and further manual curation procedures were utilised in the determination of candidate ORF features.

**Fragment Length Organisation Similarity Score Analysis**

Using the approach introduced in [14], we computed the fragment length organisation similarity score (FLOSS) for CDSs, tRNAs, ncRNAs and putative ORF features. Briefly, the FLOSS for a given genic feature is formed by calculating the sum of the absolute difference of the mean empirical distribution of ribosome profiling read lengths for all CDSs not overlapping non-coding features and the corresponding observed empirical distribution of ribosome profiling read lengths for that genic feature at a range of specified lengths.

**LC-MS tryptic peptide mapping**

Tryptic peptides were aligned to the *Mycobacterium abscessus* ATCC 19977 genome sequence (RS: NC_010397, GB: CU458896) using a database search approach implemented in PEAKS 6.0 proteomics software [15]. A parent mass error tolerance of 6.0 ppm was used in conjunction with a maximum of 2 missed cleavages along with one non-specific cleavage.

**Identification of Protein Domains**

Proteins domains for MAB_4937 were identified using SMART [16].

**Visialization Tools**

Visualisation comprising hive plot was generated using method described previously [17]. All circle plots were generated using Circos (0.67-7) [18]. The remaining visualisations were generated using ggplot2 (1.0.0) [19].

Reference List

1. Voskuil MI, Schnappinger D, Visconti KC, Harrell MI, Dolganov GM, Sherman DR *et al*.: **Inhibition of Respiration by Nitric Oxide Induces a *Mycobacterium tuberculosis* Dormancy Program.** *J Exp Med* 2003, **198:** 705-713.

2. Nash KA, Brown-Elliott BA, Wallace RJ: **A Novel Gene, erm(41), Confers Inducible Macrolide Resistance to Clinical Isolates of *Mycobacterium abscessus* but Is Absent from *Mycobacterium chelonae*.** *Antimicrob Agents Chemother* 2009, **53:** 1367-1376.

3. Bernut A, Le Moigne V, Lesne T, Lutfalla G, Herrmann JL, Kremer L: **In Vivo Assessment of Drug Efficacy against *Mycobacterium abscessus* Using the Embryonic Zebrafish Test System.** *Antimicrob Agents Chemother* 2014, **58:** 4054-4063.

4. Turner KH, Wessel AK, Palmer GC, Murray JL, Whiteley M: **Essential genome of *Pseudomonas aeruginosa* in cystic fibrosis sputum.** *Proc Natl Acad Sci U S A* 2015, **112:** 4110-4115.

5. Dinan AM, Tong P, Lohan AJ, Conlon KM, Miranda-CasoLuengo AA, Malone KM *et al*.: **Relaxed Selection Drives a Noisy Noncoding Transcriptome in Members of the *Mycobacterium tuberculosis* Complex.** *mBio* 2014, **5:** e01169-14.

6. Ingolia NT, Brar GA, Rouskin S, McGeachy AM, Weissman JS: **The ribosome profiling strategy for monitoring translation in vivo by deep sequencing of ribosome-protected mRNA fragments.** *Nat Protoc* 2012, **7:** 1534-1550.

7. Langmead B, Trapnell C, Pop M, Salzberg SL: **Ultrafast and memory-efficient alignment of short DNA sequences to the human genome.** *Genome Biol* 2009, **10:** R25.

8. Li H, Handsaker B, Wysoker A, Fennell T, Ruan J, Homer N *et al*.: **The Sequence Alignment/Map format and SAMtools.** *Bioinformatics* 2009, **25:** 2078-2079.

9. Robinson MD, McCarthy DJ, Smyth GK: **edgeR: a Bioconductor package for differential expression analysis of digital gene expression data.** *Bioinformatics* 2010, **26:** 139-140.

10. Wu S, Zhu Z, Fu L, Niu B, Li W: **WebMGA: a customizable web server for fast metagenomic sequence analysis.** *BMC Genomics* 2011, **12:** 444.

11. R Core Team (2013). R: **A Language and Environment for Statistical Computing**. 2016. R Foundation for Statistical Computing.

12. Python Software Foundation. **Python Language Reference**. <http://www.python.org>. 2016.

13. Quinlan AR, Hall IM: **BEDTools: a flexible suite of utilities for comparing genomic features.** *Bioinformatics* 2010, **26:** 841-842.

14. Ingolia NT, Brar GA, Stern-Ginossar N, Harris MS, Talhouarne GlJS, Jackson SE *et al*.: **Ribosome Profiling Reveals Pervasive Translation Outside of Annotated Protein-Coding Genes.** *Cell Reports* 2014, **8:** 1365-1379.

15. Zhang J, Xin L, Shan B, Chen W, Xie M, Yuen D *et al*.: **PEAKS DB: De Novo Sequencing Assisted Database Search for Sensitive and Accurate Peptide Identification.** *Mol Cell Proteomics* 2012, **11:** M111.

16. Mitchell A, Chang HY, Daugherty L, Fraser M, Hunter S, Lopez R *et al*.: **The InterPro protein families database: the classification resource after 15 years.** *Nucleic Acids Res* 2015, **43:** D213-D221.

17. Krzywinski M, Birol I, Jones SJ, Marra MA: **Hive plots - Rational approach to visualizing networks.** *Briefings in Bioinformatics* 2011.

18. Krzywinski M, Schein J, Birol n, Connors J, Gascoyne R, Horsman D *et al*.: **Circos: An information aesthetic for comparative genomics.** *Genome Res* 2009, **19:** 1639-1645.

19. Wickham Hadley. **ggplot2: Elegant Graphics for Data Analysis**. 2009. Springer-Verlag New York, 2009.
