# Supplementary material for: Functional characterization of the Mycobacterium abscessus genome coupled with condition specific transcriptomics reveals conserved molecular strategies for host adaptation and persistence
Source: BMC Genomics. 2016 Aug 5;17:553. doi: 10.1186/s12864-016-2868-y (PMC4974804; doi:10.1186/s12864-016-2868-y)
Supplement: Additional file 6: Figure S2. — Enrichment of functional categories among differentially expressed genes in response to antibiotic treatment. (DOCX 80 kb) [file 12864_2016_2868_MOESM6_ESM.docx]

**
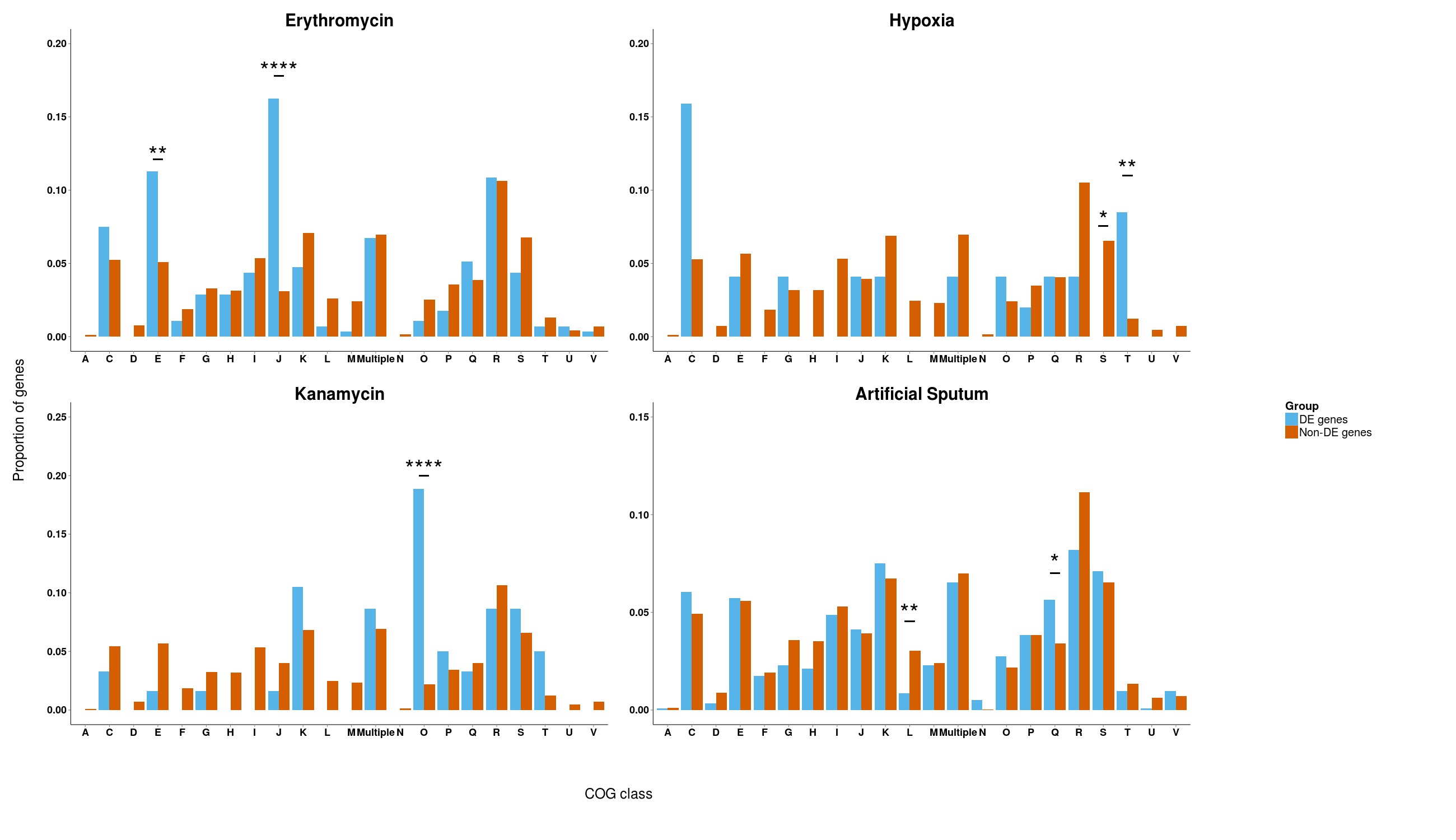
**

**Additional file 6: Figure S2. Enrichment of functional categories among DE genes in response to antibiotic treatment, hypoxia or growth in an artificial CF sputum.** *M. abscessus* genes were classified according to the Clusters of Orthologous Groups (COG) annotation scheme. Bar plots show the proportion of DE (blue) and non-DE (red) genes annotated in each category. The proportions of DE and non-DE genes in each category were compared using the Chi-Squared contingency test in Python, and multiple testing correction was carried out by implementing the Benjamini-Hochberg approach in R. Categories with a significant difference in proportional representation among DE and non-DE genes are indicated with asterisks (** FDR ≤ 0.01, **** FDR ≤ 0.0001).
COG category labels:  A = RNA processing and modification; C = Energy production and conversion; D = Cell cycle control, cell division, chromosome partitioning; E = Amino acid transport and metabolism; F = Nucleotide transport and metabolism; G = Carbohydrate transport and metabolism; H = Coenzyme transport and metabolism; I = Lipid transport and metabolism; J = Translation, ribosomal structure and biogenesis; K = Transcription; L = Replication, recombination and repair; M = Cell wall/membrane/envelope biogenesis; Multiple = Multiple classes; N = Cell motility; None = No assigned COG; O = Posttranslational modification, protein turnover, chaperones; P = Inorganic ion transport and metabolism; Q = Secondary metabolites biosynthesis, transport and catabolism; R = General function prediction only; S = Function unknown; T = Signal transduction mechanisms; U = Intracellular trafficking, secretion, and vesicular transport; V = Defense mechanisms.
