# Supplementary material for: Functional characterization of the Mycobacterium abscessus genome coupled with condition specific transcriptomics reveals conserved molecular strategies for host adaptation and persistence
Source: BMC Genomics. 2016 Aug 5;17:553. doi: 10.1186/s12864-016-2868-y (PMC4974804; doi:10.1186/s12864-016-2868-y)
Supplement: Additional file 7: Table S1. — List of genes with predicted roles in mediating antibiotic resistance which are differentially expressed upon antibiotic exposure. (DOCX 12 kb) [file 12864_2016_2868_MOESM7_ESM.docx]

**Additional file 7: Table S1.** Log fold changes (LogFC) of genes with predicted roles in mediating antibiotic resistance. Non-DE = not differentially expressed under this condition.

| **Gene** | **LogFC** | | **Function** |
| --- | --- | --- | --- |
|  | **Kanamycin** | **Erythromycin** |  |
| MAB_0163c | 1.06 | 3.38 | Probable phosphotransferase |
| MAB_0880 | Non-DE | 2.36 | hypothetical protein |
| MAB_1395 | Non-DE | 2.47 | Probable multidrug resistance transporter, Bcr/CflA family |
| MAB_1396 | Non-DE | 1.89 | Probable drug resistance transporter, EmrB/QacA subfamily |
| MAB_1409c | Non-DE | 3.11 | Putative drug antiporter protein precursor (Tap) |
| MAB_1846 | 1.37 | 2.04 | Putative ABC transporter ATP-binding protein |
| MAB_2297 | Non-DE | 2.70 | Probable methyltransferase (erm41) |
| MAB_2355c | Non-DE | 2.95 | Putative ABC transporter ATP-binding protein |
| MAB_2396 | Non-DE | 1.49 | Probable acetyltransferase |
| MAB_2622c | Non-DE | -1.05 | Branched-chain amino acid ABC transporter (LivF) |
| MAB_2780c | Non-DE | 2.13 | Putative transporter |
| MAB_2958 | -2.07 | -1.26 | Putative transmembrane-transport protein |
| MAB_2989 | Non-DE | 2.15 | Probable chloramphenicol acetyltransferase |
| MAB_3372 | Non-DE | -1.06 | Putative transcriptional regulator, TetR family |
| MAB_3891c | 1.28 | Non-DE | Probable transcriptional regulator, LuxR family |
| MAB_4395 | Non-DE | 2.00 | Aminoglycoside 2'-N-acetyltransferase |
| MAB_4687 | -1.09 | -1.24 | Putative transcriptional regulator, TetR family |
| MAB_4837c | 1.19 | 4.32 | Possible phosphotransferase |
