# Supplementary material for: Functional characterization of the Mycobacterium abscessus genome coupled with condition specific transcriptomics reveals conserved molecular strategies for host adaptation and persistence
Source: BMC Genomics. 2016 Aug 5;17:553. doi: 10.1186/s12864-016-2868-y (PMC4974804; doi:10.1186/s12864-016-2868-y)
Supplement: Additional file 8: Table S2. — List of genes encoding predicted membrane transporters which are differentially expressed upon antibiotic exposure. (DOCX 13 kb) [file 12864_2016_2868_MOESM8_ESM.docx]

**Additional file 8: Table S2.** Log fold changes (LogFC) of genes encoding predicted membrane transporters which are DE upon antibiotic exposure. Non-DE = not differentially expressed in this condition. APC = Amino Acid-Polyamine-Organocation, ABC = ATP-binding cassette, DMT = Drug/Metabolite Transporter, MFS = Major Facilitator Superfamily, MmpL = Mycobacterial membrane protein Large, NRAMP = Metal Ion (Mn^2+^-iron) Transporter, GAP = Peptidoglycolipid Addressing Protein, Rhtp = Resistance to Homoserine/Threonine

| **Gene** | **LogFC** | | **Transporter type** |
| --- | --- | --- | --- |
|  | **Kanamycin** | **Erythromycin** |  |
| MAB_3829c | Non-DE | -1.02 | APC |
| MAB_4323 | Non-DE | 1.32 | APC |
| MAB_1007c | Non-DE | -1.22 | ABC |
| MAB_1008c | Non-DE | -1.08 | ABC |
| MAB_1009c | Non-DE | -1.13 | ABC |
| MAB_1846 | 1.37 | 2.04 | ABC |
| MAB_2177 | 1.11 | 1.96 | ABC |
| MAB_2355c | Non-DE | 2.95 | ABC |
| MAB_2622c | Non-DE | -1.05 | ABC |
| MAB_4155c | -1.05 | Non-DE | ABC |
| MAB_4564c | Non-DE | -1.14 | ABC |
| MAB_4566c | Non-DE | -1.18 | ABC |
| MAB_0677c | 1.44 | 2.65 | DMT |
| MAB_0766 | 1.33 | 4.16 | DMT |
| MAB_2640c | Non-DE | 1.04 | DMT |
| MAB_3180 | Non-DE | 1.53 | DMT |
| MAB_3762 | Non-DE | 1.05 | DMT |
| MAB_0880 | Non-DE | 2.36 | MFS |
| MAB_1395 | Non-DE | 2.47 | MFS |
| MAB_1396 | Non-DE | 1.89 | MFS |
| MAB_1409c | Non-DE | 3.11 | MFS |
| MAB_2273 | 1.39 | 3.70 | MFS |
| MAB_2780c | Non-DE | 2.13 | MFS |
| MAB_2958 | -2.07 | -1.26 | MFS |
| MAB_4716c | -1.31 | Non-DE | NRAMP |
| MAB_2275 | Non-DE | 1.20 | MmpL |
| MAB_4698 | Non-DE | -1.27 | GAP |
| MAB_3913 | Non-DE | 2.53 | RhtB |
